# Diffusion MR tractography of the heart

**DOI:** 10.1186/1532-429X-11-47

**Published:** 2009-11-13

**Authors:** David E Sosnovik, Ruopeng Wang, Guangping Dai, Timothy G Reese, Van J Wedeen

**Affiliations:** 1 Martinos Center for Biomedical Imaging, Massachusetts General Hospital, Harvard Medical School, Boston MA, USA; 2 Cardiology Division, Massachusetts General Hospital, Harvard Medical School, Boston MA, USA; 3 Center for Molecular Imaging Research, Massachusetts General Hospital, Harvard Medical School, Boston MA, USA; 4 Harvard-MIT Division of Health Sciences and Technology, Cambridge MA, USA

## Abstract

Histological studies have shown that the myocardium consists of an array of crossing helical fiber tracts. Changes in myocardial fiber architecture occur in ischemic heart disease and heart failure, and can be imaged non-destructively with diffusion-encoded MR. Several diffusion-encoding schemes have been developed, ranging from scalar measurements of mean diffusivity to a 6-dimensional imaging technique known as diffusion spectrum imaging or DSI. The properties of DSI make it particularly suited to the generation of 3-dimensional tractograms of myofiber architecture. In this article we review the physical basis of diffusion-tractography in the myocardium and the attributes of the available techniques, placing particular emphasis on DSI. The application of DSI in ischemic heart disease is reviewed, and the requisites for widespread clinical translation of diffusion MR tractography in the heart are discussed.

## Introduction

The myocardium can be studied at several spatial scales. New techniques, such as molecular imaging, are providing important insights into cardiac disease at the cellular and subcellular levels [1-3]. At the other end of the spectrum, parameters of regional and whole organ function such as ejection fraction, perfusion, viability and strain are now routinely used in clinical practice [4,5]. The microstructural organization of the myocardium, however, has been less extensively studied, although changes at this scale could provide important biological insights and a mechanism linking cellular and whole-organ pathology [6-8]. Here we describe our initial ex-vivo experience with a relatively new magnetic resonance (MR) technique, diffusion spectrum MR tractography, capable of imaging myocardial fiber architecture at the microstructural level.

In a series of breakthrough histological studies, Streeter and colleagues demonstrated that cardiomyocytes form tracts with a crossing helical architecture [9,10]. Myofiber tracts in the subendocardium have a positive or right-handed helix angle, those in the mid-myocardium are circumferential and those in the subepicardium have a negative or left-handed helix angle [9,10]. These fiber tracts form laminar sheets [11-14], and it is the shear, extension, thickening and radial reorientation of these sheets that allows the myocardium to thicken in systole [12,15-17]. Changes in scalar indices of diffusion and myofiber anatomy have been documented in a variety of small and large animal models of cardiac disease [18-23], as well as in humans [24-26]. In the majority of these studies, however, fiber anatomy was visualized only at discrete points in the myocardium. In the current article we focus on the use of diffusion-encoded MR to create continuous 3-dimensional tractograms of myocardial fiber architecture. We place particular emphasis on our recent experience in the heart with diffusion spectrum MR tractography [27]. We review the rationale and theoretical basis of MR tractography, its application in animal models of ischemic heart disease, the properties of other diffusion-encoding schemes such as diffusion tensor and q-ball imaging, and the pathway towards clinical translation of MR tractography in the heart.

### Diffusion Spectrum MR

Diffusion imaging can be performed at several levels of complexity, ranging from the simple acquisition of a single diffusion-weighted image to the complex but robust acquisition scheme used in diffusion spectrum imaging (DSI) [28,29]. Diffusion tensor imaging (DTI) and q-ball imaging can be thought of as formalisms that sample diffusion or q-space with an intermediate level of complexity [28,29]. While complex, DSI is the only technique derived directly from first principles [30], is hypothesis free [28,30], broadly generalizable, and is regarded by many (including the authors) as the gold standard diffusion imaging technique [28,31-33]. In the current implementation of DSI, q-space is sampled with 515 diffusion-encoding vectors or q-vectors, although simulations containing up to 925 q-vectors have been performed [31]. The angular resolution and accuracy of DSI result in large part from the number and distribution of the samples acquired in q-space, analogous to the manner in which the region of support and sample density of k-space influence the spatial resolution and field-of-view of an image.

The q-vectors used to sample q-space in a DSI experiment vary in both their strength (b-value) and spatial orientation, sampling q-space in a dense 3D lattice. The b-value of a q-vector is proportional to the product of the square of the gradient strength and the diffusion time interval. (b ~ q^2^Δ, where q = γδG and γ is the gyromagnetic ratio, δ is the gradient duration, G is the gradient strength and Δ is the diffusion time interval). The b-value in a diffusion-encoded acquisition is in some ways analogous to the degree of velocity encoding (venc) used during phase contrast imaging. Higher b-values increase the resolution of the diffusion spectrum, and are thus desirable. However, an excessively high b-value can reduce image signal-to-noise ratio (SNR) severely, and an optimal balance between these two competing factors must thus be struck [31,33]. B-values greater than 10,000 s/mm^2 ^have been used both in-vivo and ex-vivo in the brain and myocardium [27,30,33].

In a DSI acquisition, each voxel in the spatial (x, y, z) domain of an image has its own 3D q-space associated with it. DSI images are thus 6-dimensional in which the 3 dimensions of q-space are superimposed on the 3 dimensions of image space [28,30]. Q-space is sampled in a DSI experiment by applying a B_0 _gradient field (q-vector) along a specific spatial orientation. The duration and intensity of the applied field (b-value) determines the length of the q-vector relative to the origin of q-space, while the vector direction is determined by the spatial orientation of the applied gradients. The resulting signal intensity determines the coefficient for that value of Q in each voxel. A 3D image with 96 × 96 × 96 voxels will thus have greater than 8 × 10^5 ^q-space datasets, with each dataset containing 515 coefficients.

**Figure 1:**
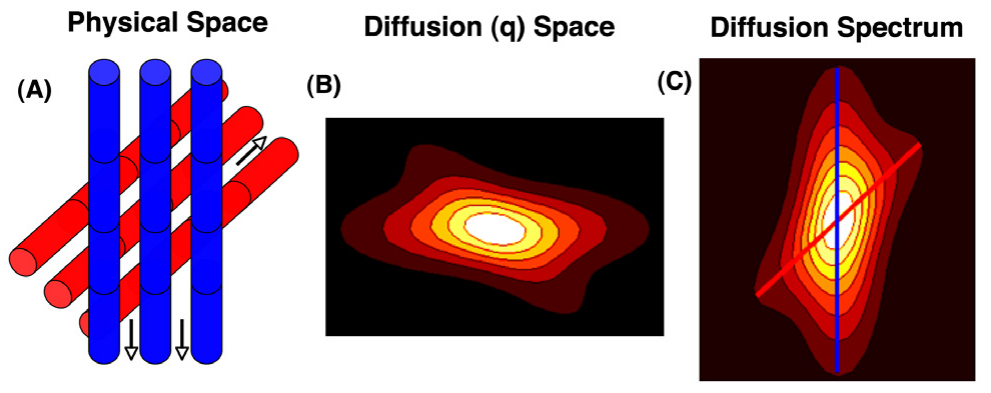
**Schematic of basic principles underlying DSI tractography**. (A) Schematic of a voxel with 2 fiber populations. Water diffuses primarily parallel to the direction of fiber orientation (arrows). (B) Each point in q-space corresponds to the signal intensity in the voxel resulting from the application of that diffusion-encoding vector. Vector length and orientation (location from the origin in q-space) are determined by the diffusion field gradient strength (intensity-time product) and the gradient direction, respectively. Signal intensity is lowest when the diffusion gradient is strongest and aligned with the fiber orientation. (C) Fourier transformation of q-space produces a probability distribution function (PDF) in which the local maxima indicate the axes of fiber orientation in that voxel (blue and red lines). Radial integration reduces the PDF to an orientation density function (ODF), which still contains the directional information needed to produce the fiber tractograms. Connection of the local maxima in the ODF of each voxel by integration into multivoxel streamlines produces the 3D myofiber tractograms.

The q-space formalism, for a single voxel and its associated q-space dataset, is depicted graphically in figure 1. The value of each coefficient in the q-space dataset of the voxel is determined by the signal intensity in the voxel during the application of the q-vector defined by that q-space coefficient. The impact of diffusion-encoding on the intensity and phase of the MR signal will be easily understood by those familiar with phase-contrast imaging in cardiovascular magnetic resonance (CMR). Velocity encoding during phase contrast CMR involves the application of two gradient lobes of equal magnitude but opposite polarity, producing a phase differential in the presence of flow. Likewise, as described by Stejskal and Tanner [34], if a pair of diffusion-encoding gradients with equal magnitude but opposite polarity is applied in the direction of diffusion, the moving spin undergoes a phase shift resulting in incomplete rephasing and attenuation of the MR signal [28,29]. Water diffuses primarily along the long axis of myofibers [35], as shown in the schematic in figure 1. The intensity of a q-space coefficient will thus be greatest when a small q-vector is directed orthogonal to the myofibers in the voxel, and lowest when a large q-vector is applied in the direction of myofiber orientation (figure 1) [28,35].

**Figure 2:**
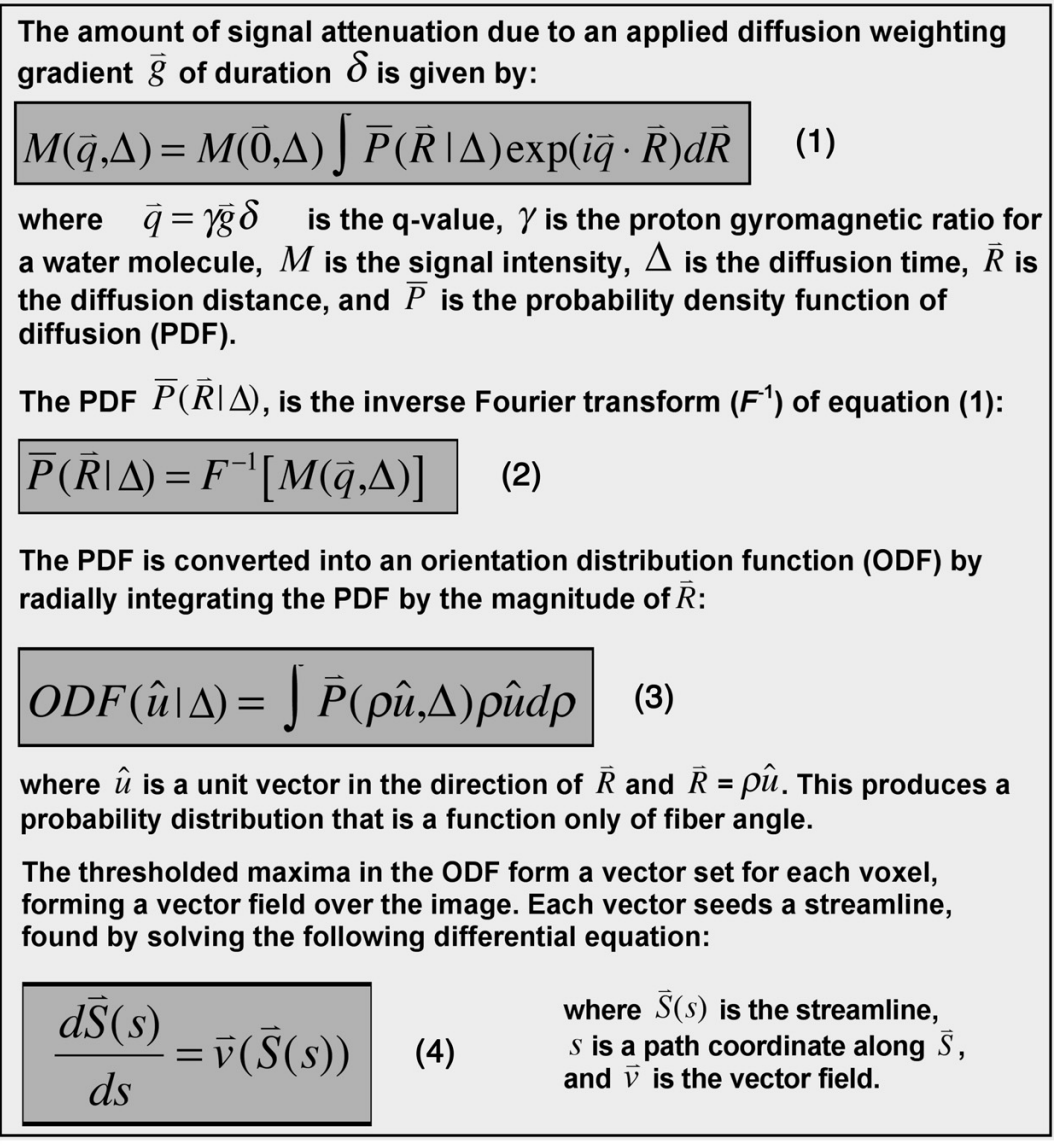
**Mathematical basis of DSI tractography**. DSI tractograms are 6 dimensional images, containing 3 dimensions of image (x, y, z) space and 3 dimensions of q-space. The generated streamlines are tangent to the ODF vector field at all points, as shown in equation 4.

Q-space and k-space are both discretely sampled Cartesian 3-spaces that are Fourier transformed to yield a useful result. While inverse Fourier transformation of k-space produces an anatomical image, the inverse Fourier transform of q-space produces a probability density function (PDF) of water diffusion (and indirectly fiber orientation) in the voxel (figure 1) [28,30]. The PDF describes the probability that a water molecule will diffuse a certain distance in a particular direction during the diffusion MR acquisition. Since only the directional information is required for DSI tractography, the PDF is usually reduced by radial integration to an orientation distribution function (ODF) [28,30]. The PDF and ODF in each voxel can have multiple local maxima, each resolving an individual fiber population in the voxel. DSI is thus able to resolve multiple fiber populations in a voxel with both high angular and spatial resolution, including crossing and converging fibers [30,33,36]. The reader interested in a more mathematical description of DSI tractography is referred to figure 2 of this article and to prior work in the field [30].

The merits and limitations of other diffusion-encoding schemes (q-ball imaging, spherical deconvolution, diffusion tensor imaging) will be discussed in detail later in the article. These techniques sample q-space less fully than DSI, enforce a diffusivity model (Funk transform, spherical harmonics, tensor) on the sampled data and degeneralize DSI to some degree. The simplest of these models that still includes directional information is DTI, but this differs however from DSI in several important ways: With DSI each distinct fiber population in a voxel is represented by a unique local maximum in the PDF/ODF [30,37,38]. As described below, however, the principal eigenvector of a diffusion tensor reflects the average direction of myofiber orientation in the voxel [38]. In addition, while the spatial resolution of DTI is determined by/identical to the resolution of the image, the 6-dimensional nature of DSI provides subvoxel resolution, determined by the resolution of the ODF [38]. The attributes of DSI are thus inherently suited to the generation of 3D tractograms of myocardial fiber architecture.

**Figure 3:**
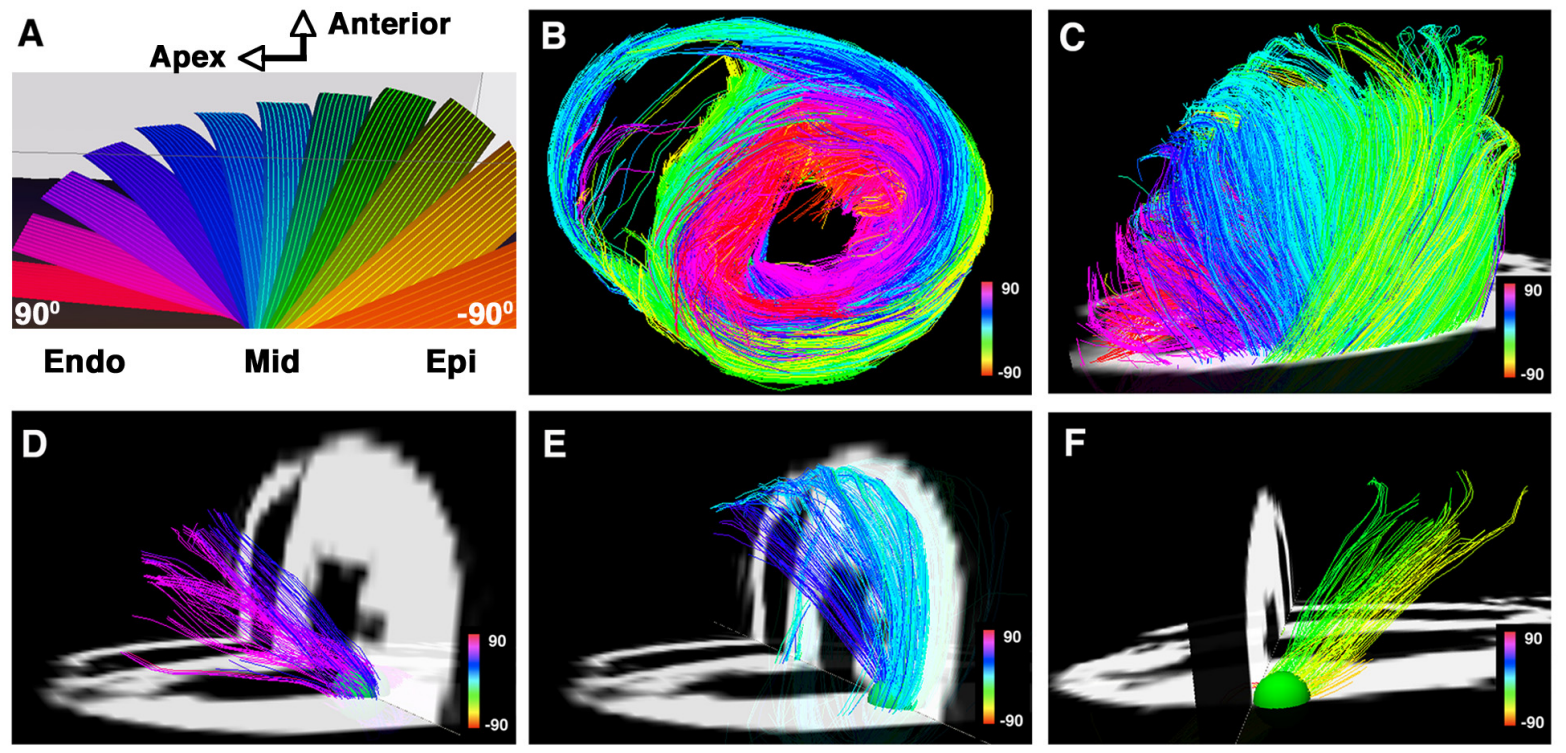
**DSI tractography of a normal rat heart ex-vivo showing the transmural variation in myofiber helix angle: The left ventricle is being viewed (A and C-F) from its lateral wall, and (B) in its short axis**. Only those fibers intersecting a spherical region-of-interest are displayed in (D-F). Subendocardial fibers have a positive or right-handed helix angle and in the lateral wall course towards the antero-apex, while those in the subepicardium have a negative or left-handed helix angle and in the lateral wall course from the antero-base towards the postero-apex. Myofiber helix angle transitions smoothly from the subendocardium to subepicardium. Fibers in the mid-myocardium have a zero helix angle and are thus circumferential. Reproduced with permission from Sosnovik et al [27].

Tractograms can be generated using deterministic or probabilistic algorithms [29,39]. Probabilistic models address the uncertainty associated with the pathways of the reconstructed tractograms, which can be significant when DTI is used [29,39]. The properties of DSI, however, provide a robust platform for the creation of deterministic myofiber tractograms since the high angular and spatial resolution of DSI significantly reduces uncertainty. Local maxima in the ODFs of each voxel form a vector field that can be integrated into streamlines or tractograms, representing pathways of maximum diffusion coherence [30,32,36-38]. The streamline will follow the path of minimal angular difference between adjacent ODFs (voxels), with a threshold angle of 18-35° used to halt further propagation of the streamline. Fiber tracts in the myocardium can be depicted in terms of their direction along Cartesian axes [40], but should optimally be depicted in terms of the helix or spiral angle they make with the long axis of the left ventricle, in accordance with the histological pattern described by Streeter [27,41,42]. DSI tractography of a normal rat heart, color coded by helix angle, is shown in figure 3[27].

### DSI Tractography of the Myocardium

**Figure 4:**
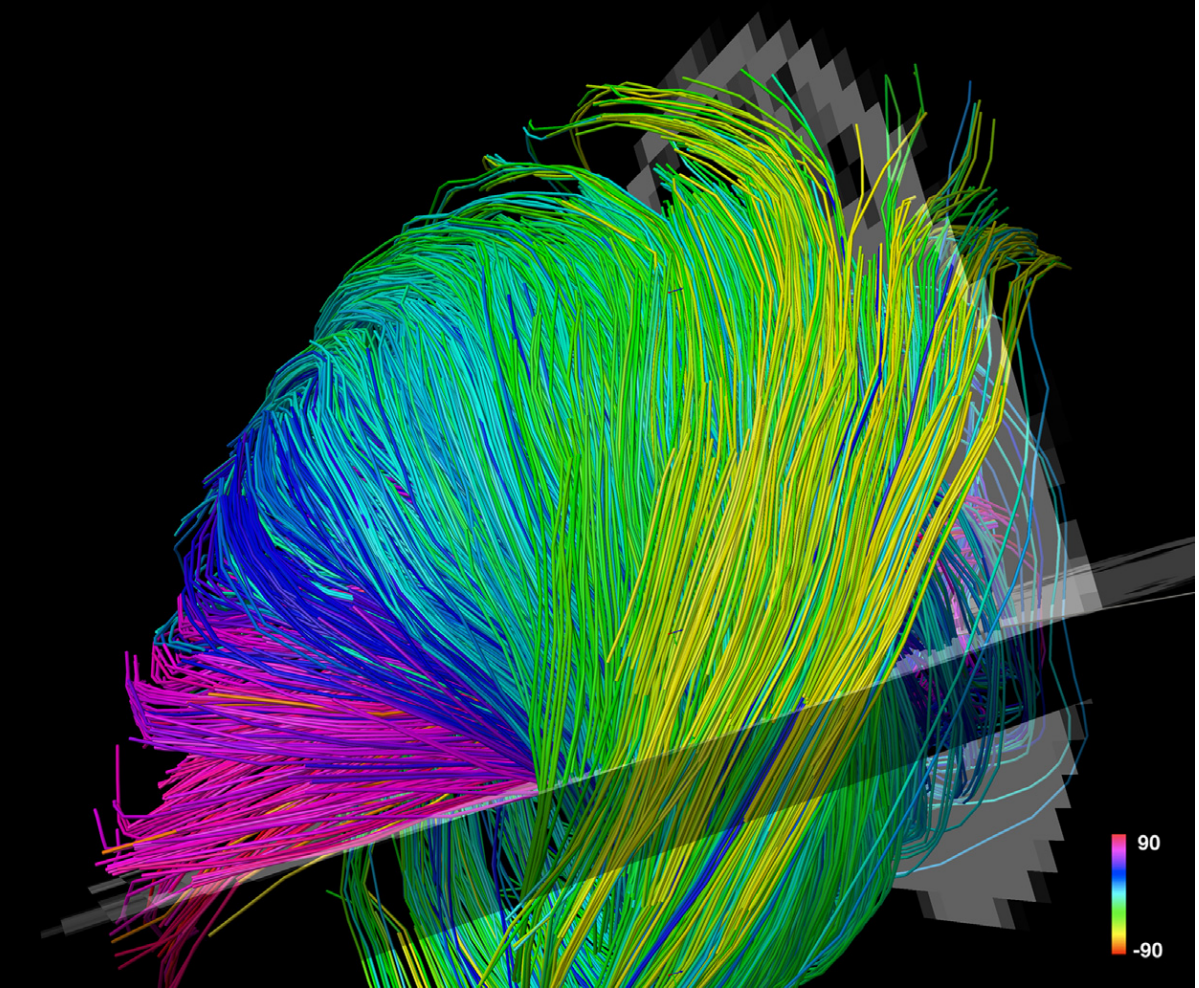
**Magnified view of myofiber tractograms generated with DSI in the lateral wall of a normal rat heart**. The crossing helical architecture of the myocardium is well seen. Myofiber tracts in the subendocardium (pink to navy-blue) and tracts in the subepicardium (green-yellow) have orthogonal helix angles and cross over each other in separate transmural planes.

**Figure 5:**
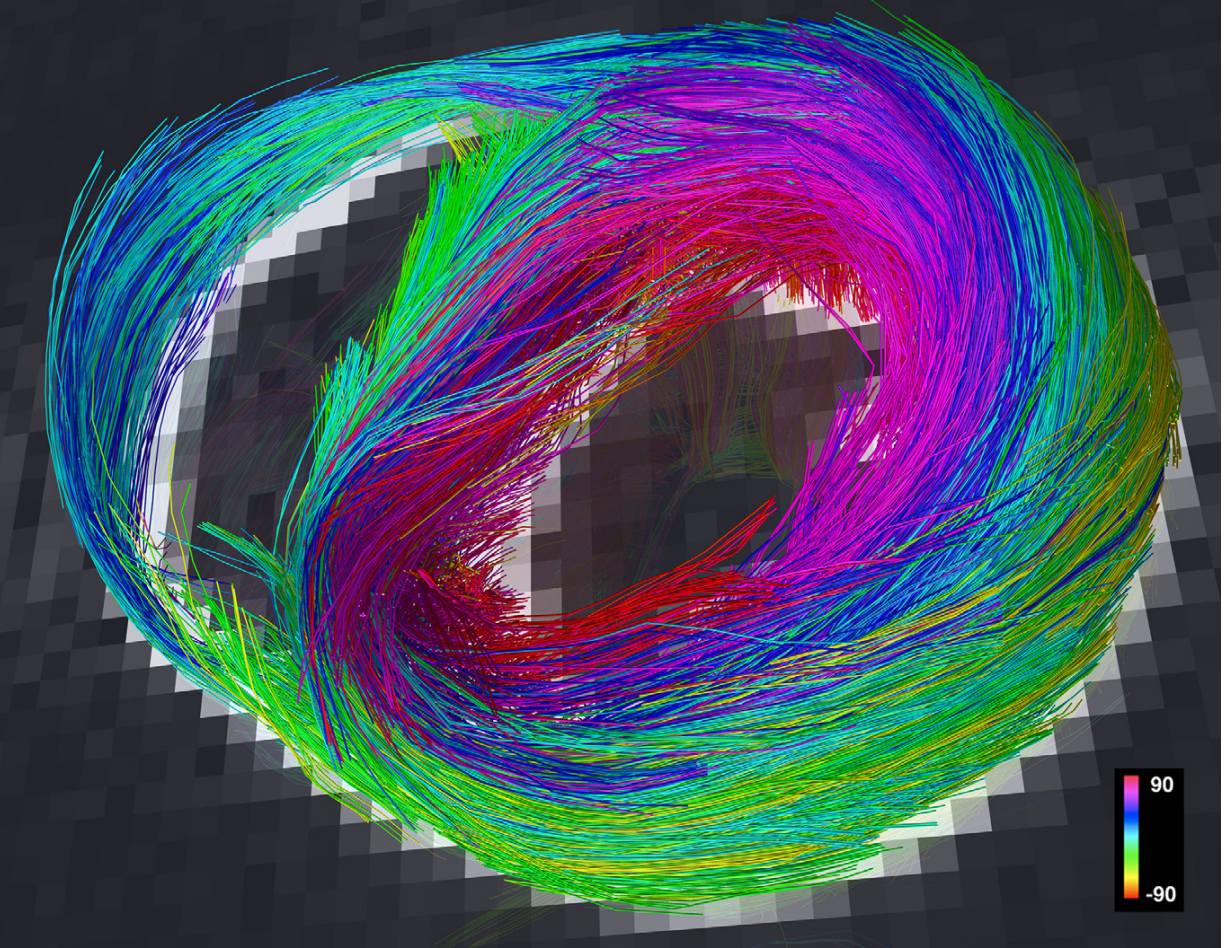
**Tomographic short axis view from a DSI dataset of a normal rat heart**. The local maxima in the ODFs provide a large number of seed points in each voxel. DSI thus facilitates the construction of fiber tractograms with a high degree of density and level of detail.

**Figure 6:**
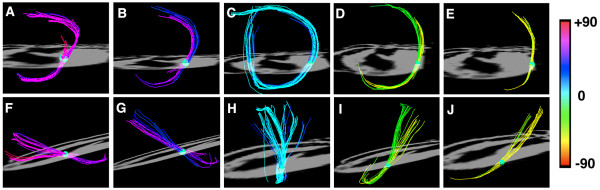
**Myofiber architecture from a DSI dataset in a normal rat heart viewed more finely using small spherical ROIs**. The heart is viewed (A-E) from the LV apex and (F-J) from the lateral wall. Myofiber tracts in both the subendocardium (A, B) and subepicardium (D, E) have similar lengths and form a half-turn of a spiral. However, the myofibers in the subendocardium of the lateral wall (F, G) course from the postero-base towards the antero-apex while the fibers in the subepicardium of the lateral wall (I, J) course from the antero-base towards the postero-apex. The myofiber tracts in the mid-myocardium (C, H) are circumferential and have a length approximately equal to the circumference of the ventricle. Reproduced with permission from Sosnovik et al [27].

The largest experience with DSI tractography in the myocardium to date has been in the imaging of normal and infarcted rat hearts ex-vivo [27]. Preliminary experience with the technique in excised hearts from mice, lambs and sheep, however, has been highly encouraging [43]. DSI tractography was able to robustly resolve the anisotropy of myocardial fiber architecture in excised fixed rat hearts [27]. As shown in figures 3, 4, 5 and 6 of normal rat hearts, the arrangement of fibers in the myocardium into an array of crossing helical structures was resolved in exquisite detail. Myofibers in the subendocardium can be seen to form a positive or right-handed helix, those in the subepicardium to form a negative or left-handed helix, and myofibers in the mid-myocardium to be circumferential (zero helix angle). The 6-dimensional nature of DSI and its subvoxel resolution resulted in dense tractographic datasets without gaps or discontinuities between adjacent myofibers [27]. The high angular and spatial resolution of DSI tractography can be fully appreciated in the magnified images shown in figures 4 and 5.

DSI tractograms can be visualized as projection images or as tomographic reconstructions of the 3D dataset. Tomographic representation involves the selection of a plane in the 3D field, the thickness of which is defined by the user. Only those myofibers that intersect the plane are shown in the reconstructed image (figures 4 and 5). The density of fibers in the myocardium, however, can make projection images and even tomographic reconstructions complex to interpret. Spherical or discoid regions-of-interest (ROIs) can thus be defined to visualize only those fiber tracts intersecting the ROI (figures 3 and 6). As shown in figure 6, myofiber tracts in the subendocardium and subepicardium form half-turns of a spiral but have orthogonal helix angles. Fibers in the subendocardium of the lateral wall track from the posterior-base to the anterior apex, while those in the subepicardium track from the anterior-base to the posterior apex [27].

**Figure 7:**
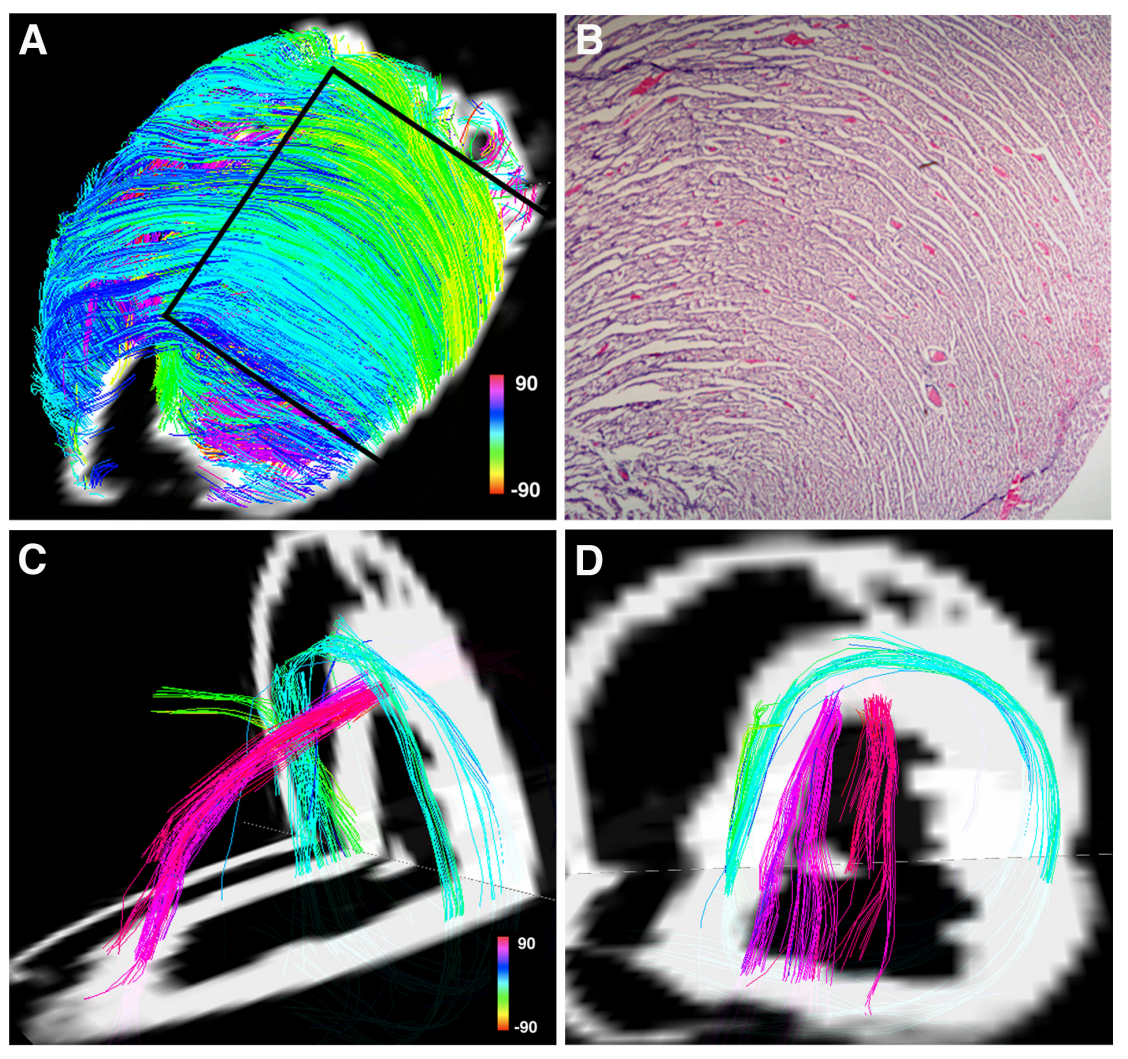
**(A) Projection DSI tractogram looking onto the anterior and anterolateral walls of a normal rat heart**. The visualized fibers have helix angles consistent with subepicardial (green-yellow) and mid-myocardial (blue) myofibers and are arranged in an orderly and dense network of myofiber sheets. (B) A hematoxylin and eosin stained section of the myocardium (outlined by the black lines in Panel A) showing a similar structure. (C, D) A normal rat heart viewed from (C) its lateral wall and (D) its apex. Subendocardial (pink) and mid-myocardial (blue) fibers cross over each other in separate transmural planes that do not intersect or make contact. Reproduced with permission from Sosnovik et al [27].

Fiber tracts in a given transmural plane in the septum have the identical helix angle to those in the lateral wall but an opposite alignment (subendocardium: anterior-base to the posterior apex and subepicardium: posterior-base to the anterior apex) and thus complete a turn of their respective helices. Throughout the myocardium a smooth evolution in myofiber helix angle was seen, as shown in figures 3 and 6. Adjacent myofiber tracts with similar helix angles could be seen to form a sheet-like structure both with DSI and histologically (figures 4 and 7) [27]. Little dispersion in myofiber helix angle was seen in a given transmural plane and in normal myocardium fibers with orthogonal helix angles made no contact and were separated from each other by myofibers with intermediate helix angles (figures 6 and 7) [27].

**Figure 8:**
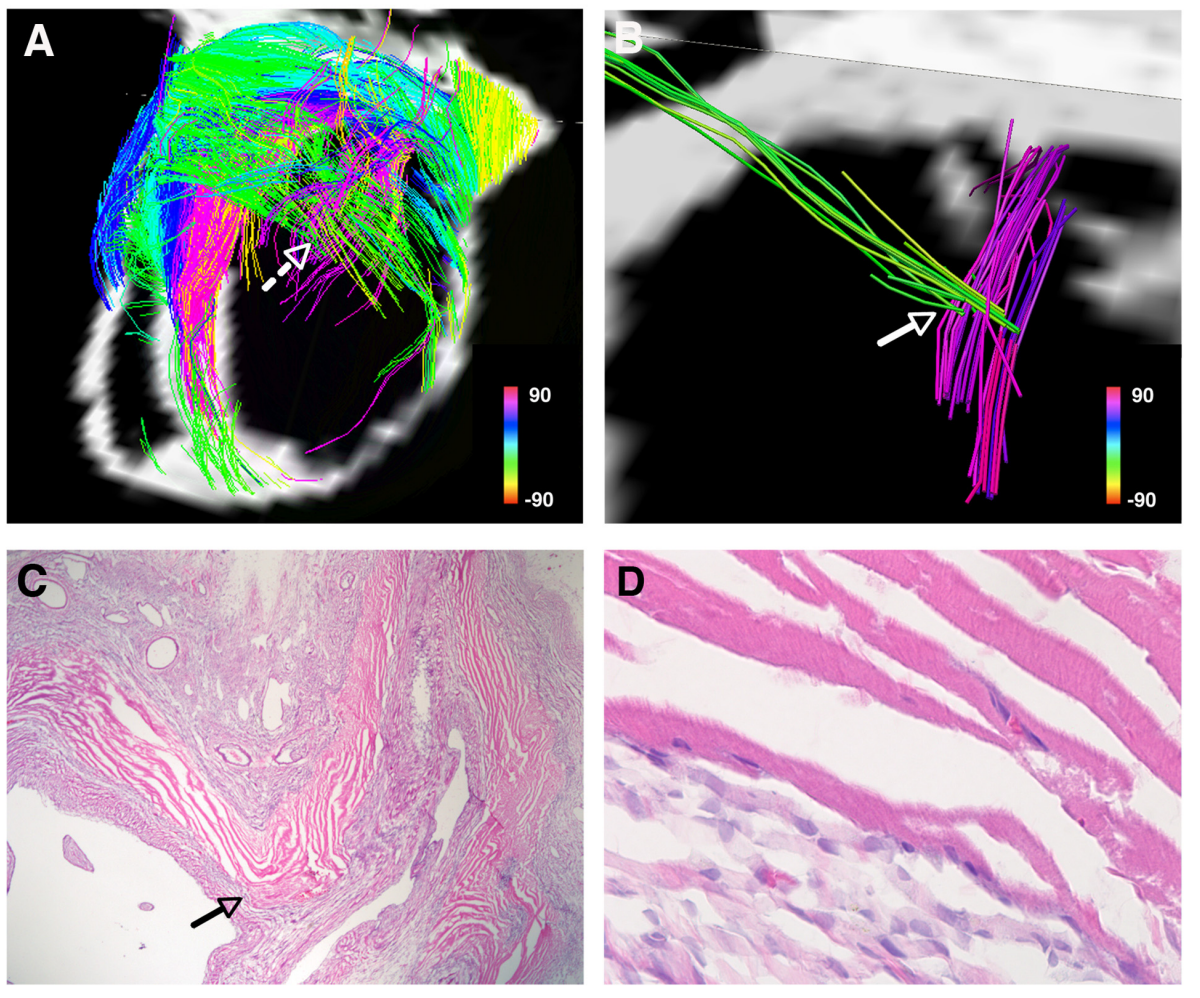
**(A) Tomographic DSI reconstruction of an infarcted rat heart**. The basal portion of the infarct and the adjacent border zones are shown. Myofiber architecture is relatively preserved in the border zones but is severely perturbed in the infarct. Numerous residual myofibers are present within the septal and basal portions of the infarct. The residual myofibers in the infarct resemble subendocardial (pink) and subepicardial (green) myofiber tracts, and make contact with/intersect each other in a mesh-like structure. The dashed white arrow marks the location of a node of orthogonal myofiber contact, which is shown in more detail in panel (B). (B) Magnified view of a node of orthogonal myofiber intersection or contact (NOMIC). (C) Hematoxylin and eosin stain (20×) of the NOMIC showing longitudinally-oriented myofibers intersecting with transversely-oriented myofibers, confirming the DSI findings. The arrows in panels (B, C) point to the area of myofiber intersection/contact. (C, D) The majority of the infarct is infiltrated with scar tissue (darker purple in color). The residual myofibers (bright pink) appear highly organized and show the striations characteristic of cardiomyocytes (panel D, 400×). Reproduced with permission from Sosnovik et al [27].

The impact of ischemic injury on myofiber architecture in infarcted rat hearts was also examined in this study [27]. The infarcted hearts were perfused-fixed with paraformaldehyde three weeks after permanent left coronary artery ligation, and imaged at 4.7 Tesla with a maximum b-value of approximately 10,000. Myocardial fiber architecture was extremely perturbed in the infarcted hearts (figure 8). However, in all cases a large number of residual myofibers were seen within the infarcts, particularly in the most basal portions of the infarct [27]. We hypothesize that these residual myofibers persist within the infarct due to pre-existing collateral networks, but this will require further study in a range of animal models.

**Figure 9:**
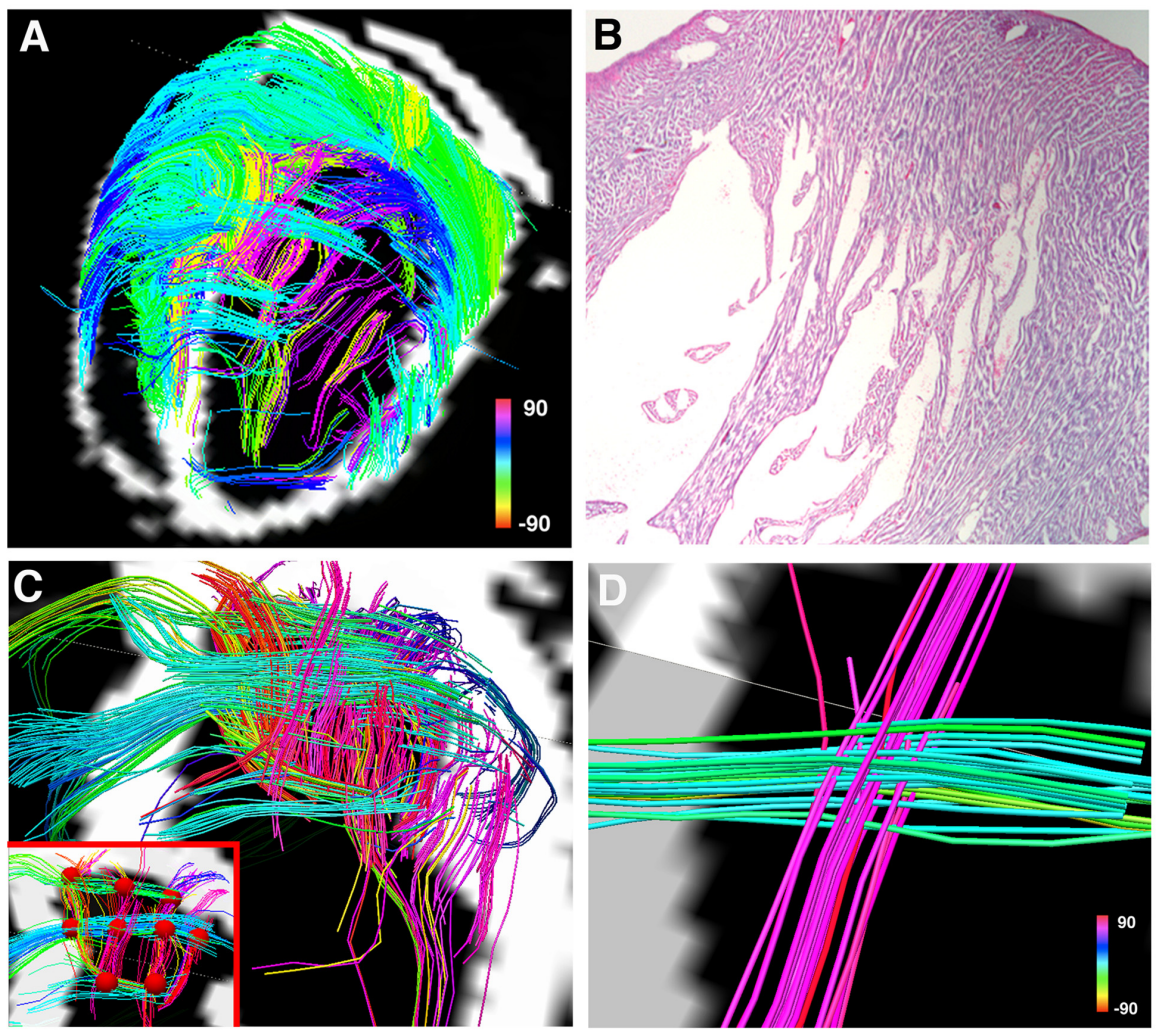
**(A) DSI tractography of an infarcted rat heart: The infarct boundary is highly irregular and characterized by numerous residual myofibers extending from within the infarct to the basal and septal border zones**. Subendocardial-like myofibers (pink) extend from the infarct to its basal border zone and form a network of orthogonal myofibers with the transversely oriented mid-to-subepicardial fibers (blue-green) that course from the infarct to the septum. (B) Hematoxylin and eosin section of the basal and lateral portion of the infarct. (C) Magnified view of the basal portion of the infarct: Residual orthogonal myofibers lie in the same plane, are not separated by an intervening layer of myofibers and can thus form nodes of orthogonal myofiber intersection or contact (NOMIC). (C) NOMICs in the infarcted myocardium (red spheres with a radius of 1 voxel, volume of 0.27 mm^3 ^and containing a pair of orthogonal myofibers) are shown in the inset at the bottom left of the panel. (D) Magnified view of a NOMIC from panel (C) showing a pair of intersecting orthogonal myofibers in more detail. Reproduced with permission from Sosnovik et al [27].

The residual myofibers within the infarcts showed several interesting microstructural properties. Many of the residual myofibers had helix angles consistent with subendocardial fibers (figures 8 and 9). Moreover, the residual subendocardial myofibers frequently made contact with residual mid and subepicardial myofibers in the infarct (figures 8 and 9) [27]. A wide dispersion in helix angles was seen in the residual fibers within the infarct. Moreover, presumably due to thinning and expansion of the infarct, residual myofibers with orthogonal helix angles frequently came into contact with one another (figures 8 and 9) [27]. Nodes of orthogonal myofiber intersection were also seen in the infarct, presumably in areas with severe local deformation in myofiber anatomy (figure 9). The residual myofibers within the infarct thus frequently formed a mesh-like structure, producing nodes of orthogonal myofiber intersection or contact (NOMIC) within the infarct [27]. The presence of similar networks of residual myofibers will need to be confirmed in other animal species as well as in humans. Further study will also be needed to determine the mechanical and electrophysiological implications of these networks of residual myofibers and the nodes of orthogonal myofiber contact within them.

### Diffusion Tensor MR (DTI)

**Figure 10:**
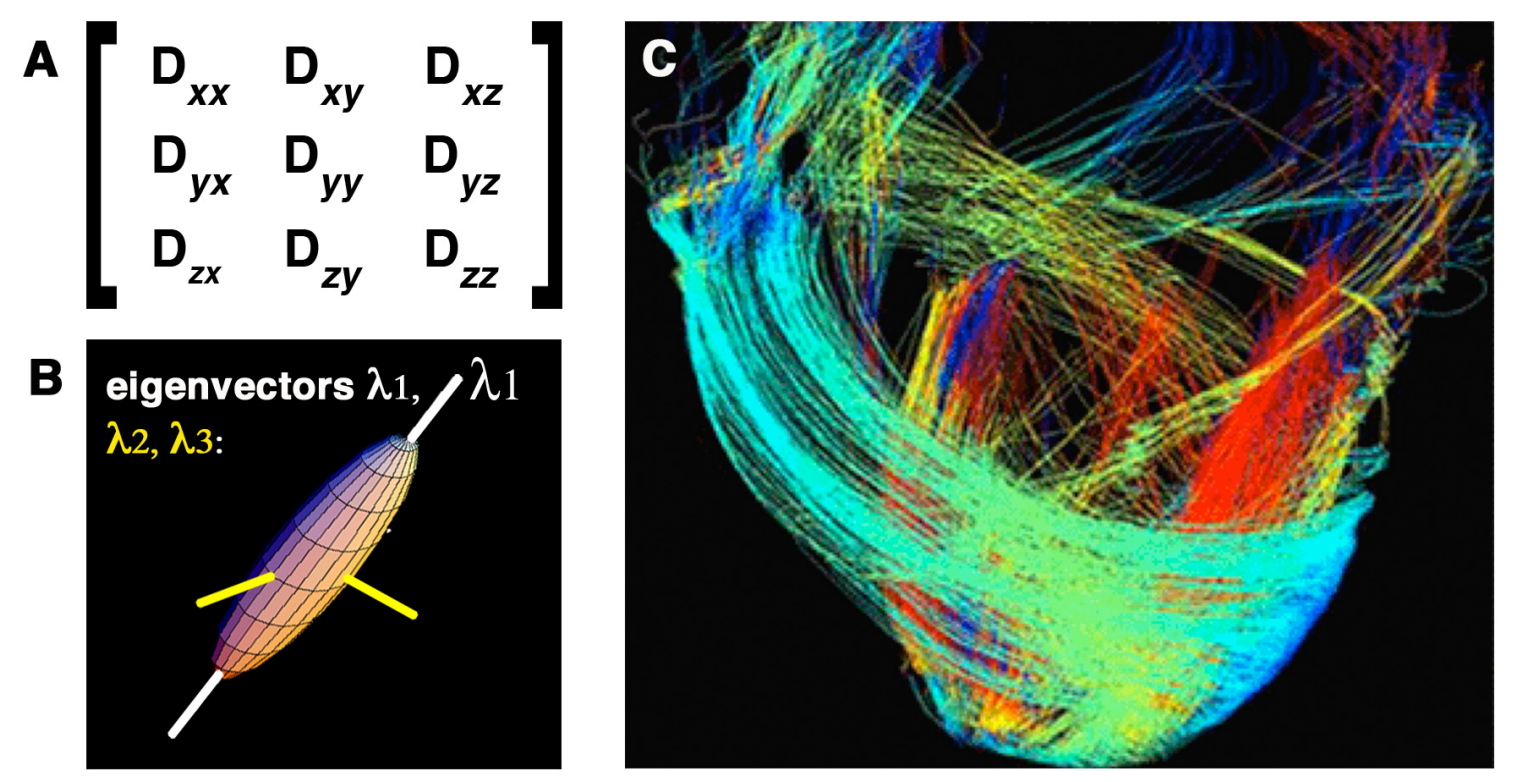
**Diffusion tensor MRI tractography**. (A) The 3 × 3 Diffusion tensor: The off-diagonal elements of the tensor are equal (D_xy _= D_yx_, D_xz _= D_zx_, D_yz _= D_zy_). (B) Diagonalization of the tensor produces three eigenvectors (λ_1_, λ_2_, λ_3_). The principal eigenvector (λ_1_) is the largest of these and describes the direction of diffusion along the long axis of muscle fibers (white axis). The principal eigenvector in a voxel, however, is a measure of the average direction of diffusion in that voxel and cannot resolve the individual directions of more than one fiber population in the voxel. (C) The principal eigenvectors in adjacent voxels can be linked to form streamlines/tractograms. DTI-tractograms, however, are less robust than those produced by DSI because only one average vector and seed point (the principal eigenvector) is present per voxel. Panel C, adapted with permission from Helm et al [41].

DTI is a formalism that samples q-space more rapidly than DSI but is based on the assumption that the diffusion in a voxel is Gaussian [28,29]. This assumption holds true when a voxel contains only a single myofiber population but introduces bias and uncertainty when this is not the case. Several useful scalar parameters of diffusion in the myocardium can be derived from DTI [18-23,28,29], but the technique is less suited to the generation of tractograms than DSI [28,33,38], as discussed below. The diffusion tensor is a symmetric second order (3 × 3) tensor (figure 10). Values along the diagonal (D_*xx*_, D_*yy *_and D_*zz*_) describe the degree of diffusion along the principal laboratory axes (x, y, z). Off-diagonal elements of the tensor describe the degree of correlation between diffusion in two directions. The nature of diffusion results in the tensor being symmetric and the transpose of the tensor is thus identical to the original tensor (alternatively stated the off-diagonal elements above and below the diagonal are equal to each other). The 3 × 3 diffusion tensor thus contains 6 independent values, and requires diffusion encoding to be performed in a minimum of 6 independent (non-linear) directions. DTI can thus be viewed conceptually as a linear approximation of DSI, in which q-space is sampled with 6 rather than 515 q-vectors [28].

Diagonalization of the diffusion tensor rotates its axes out of the laboratory frame (x, y, z) and along the eigenvectors of the tensor, which reflect the diffusion properties of the tissue in the voxel (figure 10) [28,29]. The principal or largest eigenvector, designated λ_1_, describes the direction of diffusion along the long axis of the myofibers in the voxel, the second largest eigenvector the direction of the myofiber sheets and the third eigenvector the direction normal to the myofibers [14,16,35]. Several important scalar parameters, such as mean diffusivity and fractional anisotropy, can be derived from the eigenvalues associated with these eigenvectors [28,29]. Studies in animals and humans have shown that both mean diffusivity and fractional anisotropy change significantly in infarcted and healing myocardium [18-26].

The principal eigenvector of the tensor can be used to estimate the average direction and helix angle of the myofibers in a voxel [14,35]. A reduction in right-handed (subendocardial) fibers and an increase in left-handed (subepicardial) fibers has been noted in animals and patients with myocardial infarction [22,25]. While of substantial value, several significant limitations of DTI impact cardiac tractography and merit discussion. DTI tractography can only resolve one fiber population, described by the principal eigenvector, in a given voxel. Moreover, the principal eigenvector is a composite measure of mean diffusion in the voxel. If a voxel contains more than one fiber population the principal eigenvector will represent the average direction of diffusion in the voxel, which may actually not be an accurate representation of the fibers in the voxel [28,39,44]. In the context of tractography, DTI thus reduces the information contained in the PDF into a single average value, the principal eigenvector. DTI can thus be viewed conceptually as a linear approximation of the displacement spectrum.

The principal eigenvectors in adjacent voxels can be connected using several algorithms to form streamlines of fiber tracts (figure 10) [39,44]. DTI tractograms, however, are limited in both angular and spatial resolution and are susceptible to a degree of bias and uncertainty introduced by the undersampling of q-space [39,44]. Because only one average fiber population per voxel can be resolved with DTI, it is unable to detect complex and converging fiber anatomy [33,38]. The lower number of seed points produced with DTI also leads to smaller and less complete tractography datasets than DSI. In theory, depending on the number of fiber populations per voxel, dramatic improvements in the spatial resolution of DTI could produce a tractographic dataset similar to DSI. In practice, however, this cannot be done because DTI acquisitions are already highly SNR constrained due to the phase dispersion induced by the diffusion-encoding gradients. A simple isotropic doubling of the resolution of a DTI acquisition, for instance, would require 64 signal averages to maintain SNR. DTI acquisitions with near microscopic resolution would thus require prohibitively long scan times even ex-vivo to maintain adequate SNR. The longer readout duration of these ultra-high resolution scans would also increase the TE and thus reduce SNR even further. The parameters of a diffusion-encoded acquisition, particularly in-vivo, are thus frequently dominated by the need to achieve adequate SNR, as discussed below.

### In-Vivo Diffusion Imaging

Diffusion, strain and velocity encoded MR are all displacement encoding techniques that rely on the presence of a residual phase following the application of equal and opposite gradients. The spatial scale of the displacement due to diffusion, however, is far lower and requires significant modifications to be made in the acquisition scheme and/or the strength of the applied gradients. Diffusion encoded acquisitions are usually performed with single-shot readouts such as EPI (echoplanar MRI) or HASTE (half Fourier acquired single shot turbo spin echo) [44], ensuring all lines of k-space have the same phase. Multishot diffusion imaging has been performed but requires a scheme to detect and correct random shot-to-shot phase changes produced by motion and the diffusion-encoding gradients [45]. The incorporation of diffusion encoding in to a HASTE sequence violates the CPMG condition since δ, and hence the time between the 90° and the first 180° refocusing pulse, is significantly greater than half the time between successive 180° refocusing pulses in the readout. This causes phase cancellation between echoes and poor image quality. Experimental techniques have been developed to address phase incoherence in diffusion-encoded HASTE, but many of these eliminate 50% of the signal (either even or odd echoes) and thus suffer from low SNR [46]. Despite the potential for susceptibility, chemical shift and ghosting artifacts to occur, the vast majority of diffusion-encoded acquisitions are thus performed with single-shot EPI.

**Figure 11:**
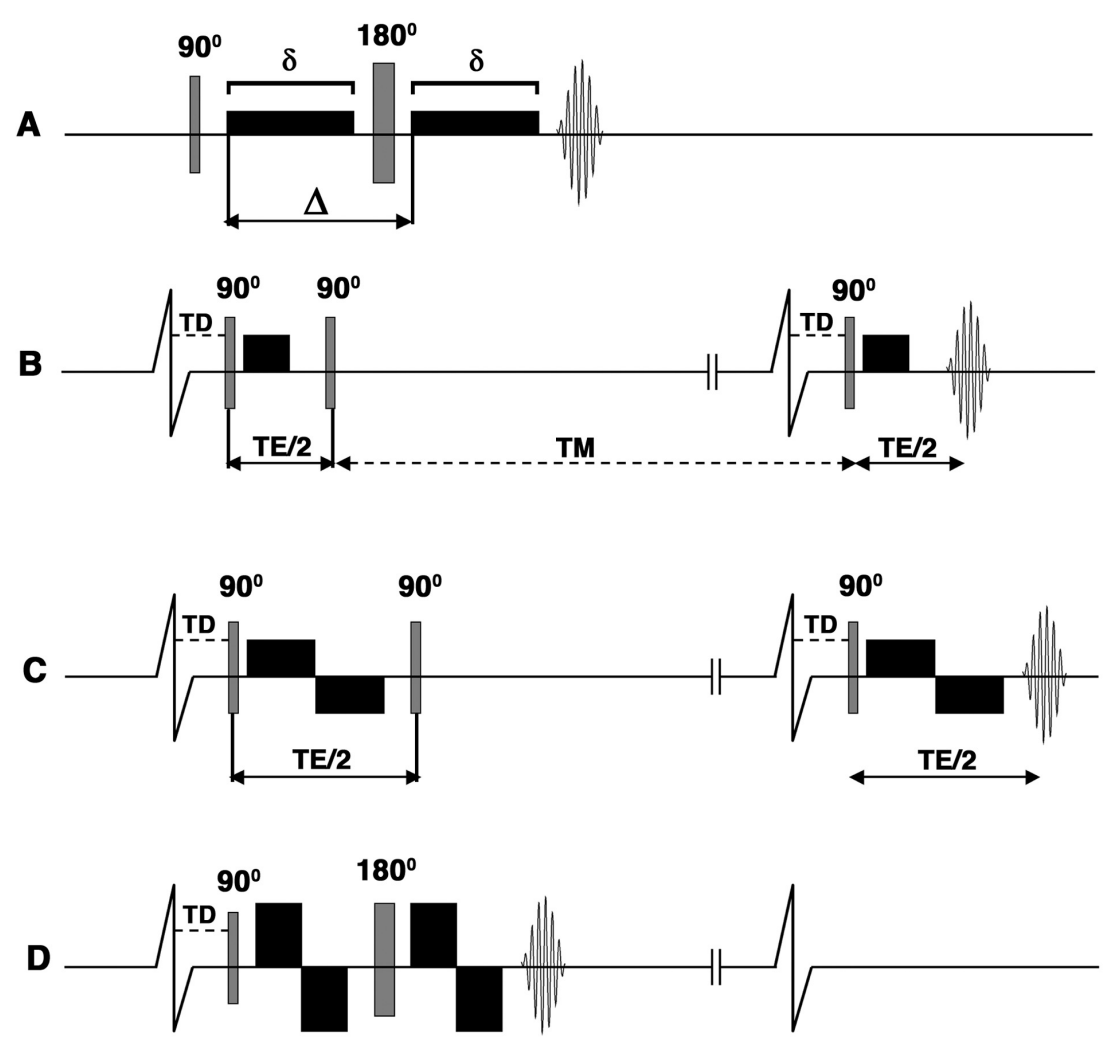
**Pulse sequences used for diffusion imaging of the myocardium**. The diffusion-encoding gradients are represented by the black rectangles, TD is the trigger delay, δ describes the duration of the diffusion-encoding gradient and Δ the diffusion time interval. Single-shot EPI readouts are used in all cases. (A) In the Stejskal-Tanner sequence diffusion-encoding gradients are placed on either side of a 180° refocusing pulse. This sequence can be used to image the myocardium ex-vivo but cannot be used to image moving tissues. (B-D): Cardiac gated sequences. The two vertical lines breaking the baseline of the ECG indicate that the timeline of the diffusion-encoding gradients is not drawn to the same scale as the ECG. (B) Diffusion-encoded stimulated echo sequence used to image the myocardium in-vivo [47]. The diffusion time (Δ) equals TE/2 plus the mixing time (TM) and is thus much longer than TE. (C) Stimulated echo sequence with bipolar diffusion-encoding gradients [12]. No diffusion encoding occurs between the second and third RF pulses with this sequence. With standard gradient strengths, the time needed to achieve an adequate b-value with the bipolar gradients lengthens the TE significantly and becomes limiting. (D) Modified (flow-compensated) Stejskal-Tanner sequence with bipolar diffusion-encoding gradients. Implementation of this sequence in-vivo was feasible on a clinical 3T scanner with gradient strengths greater than 80 mT/m [52].

The potential resolution of a single-shot EPI readout in a stationary tissue such as the brain is determined by the T2 of the tissue (which limits the TE that can be used) and the overall time available for image acquisition. EPI of the heart, however, must also contend with susceptibility artifacts from the lungs, the high fat content in the chest wall and a finite acquisition window in which image quality is not degraded by motion. Despite the use of parallel imaging and multi-element arrays, the spatial resolution of single shot EPI in the heart is limited. This places a fundamental limit on the resolution of DTI, which has the identical resolution to the EPI image. Diffusion-encoding is extremely sensitive to bulk motion [47], which in the heart is significantly greater than the motion of water due to diffusion. Diffusion-encoded sequences in the heart must thus be designed to be insensitive to bulk cardiac motion as well as myocardial strain in order to be performed in-vivo [47-49]. Several approaches have been described to accomplish this (figure 11), depending in large part on the gradient strength of the clinical scanner.

Tractography of stationary structures such as the brain and the myocardium ex-vivo can be performed with a Stejskal-Tanner diffusion encoded sequence [28,29,34]. In practice, however, diffusion-encoded imaging of the brain is frequently performed with a twice refocused spin echo sequence to limit the effects of eddy currents on image quality [50]. The diffusion-encoding gradients in the Stejskal-Tanner sequence are placed on either side of a 180° refocusing pulse [28,29,34], which refocuses the diffusion-encoded signal at the echo time of the spin echo EPI readout (figure 11A). The use of this sequence in the heart in-vivo, however, is precluded by motion. A stimulated echo approach has thus been developed to overcome this (figure 11B) [47]: Three 90° radiofrequency (RF) excitation pulses are applied within two successive RR intervals. The first and third excitation pulses have identical trigger delays from the onset of the successive R-waves and are both followed by unipolar diffusion encoding gradients. The second 90 degree RF pulse is placed a duration of TE/2 from the first, and effectively flips the transverse magnetization back into the longitudinal plane, where it is subject to R1 decay but not to R2* decay and motion induced phase change. The diffusion sensitivity of the sequence is determined by the physical displacement of water between the onset of the first and second unipolar diffusion encoding gradients. Diffusion sensitization thus continues to occur while the magnetization produced by the first excitation pulse is stored and protected in the longitudinal axis during a period known as the mixing time (TM). The application of the third 90-degree RF pulse and the second unipolar gradient produce a diffusion-encoded stimulated echo that has adequate diffusion sensitization and is largely free of motion related artifacts [47].

Several caveats of this approach, however, need to be considered. Diffusion imaging is frequently signal-to-noise (SNR) constrained and a stimulated echo EPI sequence has half the SNR of a spin-echo EPI readout. In addition, the delay between the R wave and the first and third RF pulses needs to be carefully selected to eliminate the effects of myocardial strain on the diffusion measurements [51]. Diffusion measurements (particularly the second and third eigenvectors) with this technique may thus be optimally made only at certain "sweet spots" in the cardiac cycle, for instance at midsystole, where the strain effects become negligible [51]. To overcome this limitation myocardial strain data can be acquired with the diffusion MR data and be used to retrospectively correct the diffusion measurements [48,49]. A strain-insensitive stimulated echo sequence has also been developed and uses a pair of bipolar diffusion-encoding gradients, rather than unipolar diffusion-encoding gradients (figure 11C) [12]. Diffusion encoding with this sequence, however, occurs only during the duration of the 2 pairs of diffusion encoding gradients, which are insensitive to first order motion terms (flow compensated) [12]. While this sequence is strain-insensitive and can be performed at any stage of the cardiac cycle, the gradient duration required for adequate diffusion encoding (b-values) significantly lengthens the TE and reduces SNR.

The stimulated echo approaches described above have been successfully used to perform DTI in patients in-vivo [24-26], but on a limited scale in a few centers of expertise. More widespread clinical performance of DSI tractography in the heart will require several technical and scientific advances to be made. Gradient technology on whole body MR scanners will need to be improved by at least a factor of 2. The potential of improved gradient performance was demonstrated recently by Gamper and colleagues on an 3 Tesla clinical system equipped with an 87 mT/m gradient [52]. The strength of this gradient allowed a spin-echo EPI readout to be used, avoiding the SNR penalty of a stimulated echo approach. A pair of bipolar diffusion encoding gradients was placed on either side of a 180° refocusing pulse in a flow-compensated modification of the Stejskal-Tanner approach (figure 11D) [52]. While the use of this approach does impose certain constraints, it overcomes several factors impeding the performance of diffusion tractography in-vivo. Clinical translation will also require improved techniques for whole-heart imaging to be developed. The development of multi-element arrays, including a recently developed 128-element cardiac array [53], has the potential to facilitate the acquisition of volumetric whole-heart datasets in a single breathold. In addition, improvements in radiofrequency and navigator technology will also facilitate the acquisition of volumetric diffusion encoded data of the heart in-vivo [54].

### Q-Space Sampling In-Vivo

Several q-space sampling schemes have been proposed for tractography of the brain in-vivo [30,31,55,56]. The optimal q-space sampling scheme for in-vivo tractography of the heart, however, needs to be considered in the context of the SNR and motion-imposed constraints specific to the heart. Nevertheless, the experience in the brain with different q-space sampling schemes remains highly relevant to in-vivo tractography of the myocardium and worthy of discussion. Diffusion tractography in the brain is SNR constrained [39,44]. Thus, even if only 6 independent diffusion encoding vectors are applied in order to perform DTI tractography, several signal averages need to be performed to achieve adequate SNR. This, however, is being less frequently done [44]. Rather than averaging data produced by the same diffusion-encoding vector several times, the scan time is used to acquire data in more than 6 directions [44,57-59]. The improvement in SNR, which is dependent on the total number of acquisitions, is similar with the two approaches. However, the accuracy of the data derived from the tensor is improved when a greater number of directions are sampled [57-59].

A voxel containing n individual myofiber populations will have 3n degrees of freedom, and likely require up to 6n independent q-vectors to fully resolve diffusion in the voxel. Whether the application of greater than 6n q-vectors would be desirable would depend in large part on the SNR of the data. The application of additional q-vectors would consume time but on the other hand increase the SNR of the image and also potentially de-alias the PDF/ODF. The more densely q-space is sampled the larger the PDF/ODF becomes, reducing the potential for aliasing. (This is analogous to increasing the FOV of an image to reduce aliasing in the spatial domain.) The approach favored by many, including ourselves, is thus to sample q-space as densely as possible within the limitations imposed by acquisition time, patient tolerance and the pulse sequence considerations discussed above.

Several high angular resolution diffusion imaging (HARDI) techniques have been developed that sample q-space more densely than DTI, but less so than DSI [55,56,60]. All of these techniques are based on certain hypotheses and assumptions, but are easier to implement in-vivo than DSI and may be well suited to imaging of the myocardium under certain scenarios. Q-ball imaging for instance involves the use of q-vectors that all have an identical and fairly large b-value [28,56,60]. Rather than sampling a 3D lattice in q-space, the technique samples the surface of a sphere with a given radius in q-space. The technique is simpler and more rapid than DSI but assumes that the selected b-value is optimal for the detection of all fiber or nerve tracts in the tissue, which may not always be the case. In normal myocardium the myofiber tracts have reasonably similar lengths and morphology (figure 6) and q-ball imaging may perform very well in this scenario. Infarcted myocardium, however, much like the brain has a highly heterogeneous population of myofibers, which may not all be detected optimally at the selected b-value. (A useful analogy to consider is the use of a single preset velocity encoding gradient to image several hemodynamic jets, despite large potential variations in the velocities of these jets). Nevertheless, q-ball imaging maintains many of the attributes of DSI, is easier to implement clinically and has the potential to be of significant value in the myocardium.

The accuracy of q-ball imaging and other heuristic methods will need to be validated against DSI tractography, which should be considered the reference gold-standard approach. Several approaches have now been used to validate the accuracy of DSI tractography ex-vivo (Table 1) and provide a solid basis for the translation of the technique. DSI tractography of the heart, tongue and brain ex-vivo has consistently resolved the fiber/tract patterns known to occur in normal organs [27,33,36]. In infarcted rat hearts a high degree of correlation has been seen between fiber architecture by DSI tractography and by histology [27]. In the brain autoradiography and manganese enhanced MR have confirmed the accuracy of DSI tractography [37,61], and the technique has been correlated with two-photon microscopy in the tongue [62]. A technique to validate DSI tractography in-vivo by detecting excess noise and low confidence in the dataset has also recently been developed [63]. The technique involves the random reshuffling of the voxel ODFs and the generation of tractograms from the scrambled vector field. The comparison of parameters in the tractographic datasets generated from the original and the reshuffled ODF vector fields provides an index of image noise and fidelity [63]. Edge weight (link between two nodes in a neuro-imaging dataset) has been used as a parameter of image confidence in the brain, and analogous parameters will need to be developed to assess the impact of noise on the accuracy of in-vivo tractography datasets in the heart. As with all new technologies, however, the ultimate validation of diffusion-tractography in the heart will be determined by its ability to influence hard clinical endpoints in cardiovascular medicine.

**Table 1:** Validation of DSI Tractography.

	Organ	Known Anatomical Features
Wedeen et al [33]	Brain	Crossing nerve tracts (optic chiasm, brainstem and others)

Gilbert et al [36]	Tongue	Core of crossing fiber tracts

Sosnovik et al [27]	Myocardium	Array of crossing helical myofibers

		

	**Organ**	**Validation Technique**

		

Lin et al [37]	Optic tracts	Manganese-enhanced MRI

Schmahmann et al [61]	Brain	Autoradiography

Gaige et al [62]	Tongue	Two-photon microscopy

Sosnovik et al [27]	Myocardium	Histology

The technique has been validated in several organ systems by resolving known anatomical features and also by direct comparison with a second imaging modality. A partial list of the performed validation studies is provided.

## Conclusion

The potential of diffusion-tractography, and in particular DSI tractography, to resolve microstructural fiber anatomy in the heart has been demonstrated in several studies ex-vivo. At present, hardware limitations on most clinical scanners constitute the principal impediment to the clinical translation of diffusion tractography in the heart. Progress in the field will thus be rapidly accelerated by the widespread introduction of clinical scanners with gradients greater than 80 mT/m. Advances in RF technology, multi-element arrays, navigators and parallel acquisition schemes will also facilitate the clinical translation of more advanced q-space acquisition schemes. Clinical translation of this promising technology, however, will be a major challenge, requiring an excellent level of collaboration between engineers, industry and physicians. Diffusion tractography does not involve ionizing radiation or exogenous contrast media, and poses no risk to the patient. The technique images the myocardium at the microstructural scale and provides information that is highly complementary to that provided by other modalities and imaging techniques. Diffusion tractography of the myocardium expands the breadth and scope of cardiovascular magnetic resonance and has the potential to become an extremely powerful tool in both the research and clinical settings.

## Competing interests

The authors declare that they have no competing interests.

## Authors' contributions

All authors were involved in the previous acquisition of data used in this review. All the authors wrote and/or edited the current article.
